# Prevalence of bortezomib-resistant constitutive NF-kappaB activity in mantle cell lymphoma

**DOI:** 10.1186/1476-4598-7-40

**Published:** 2008-05-19

**Authors:** David T Yang, Ken H Young, Brad S Kahl, Stephanie Markovina, Shigeki Miyamoto

**Affiliations:** 1 Department of Pharmacology, University of Wisconsin School of Medicine and Public Health, Madison, USA; 2 Department of Pathology and Laboratory Medicine, University of Wisconsin School of Medicine and Public Health, Madison, USA; 3 Department of Medicine, University of Wisconsin School of Medicine and Public Health, Madison, USA; 4 Paul P. Carbone Comprehensive Cancer Center, University of Wisconsin, Madison, USA; 5 Program in Cellular and Molecular Biology and Medical Scientist Training Program, University of Wisconsin School of Medicine and Public Health, Madison, USA

## Abstract

**Background:**

The proteasome inhibitor bortezomib can inhibit activation of the transcription factor NF-κB, a mechanism implicated in its anti-neoplastic effects observed in mantle cell lymphoma (MCL). However, NF-κB can be activated through many distinct mechanisms, including proteasome independent pathways. While MCL cells have been shown to harbor constitutive NF-κB activity, what fraction of this activity in primary MCL samples is sensitive or resistant to inhibition by bortezomib remains unclear.

**Results:**

Proteasome activity in the EBV-negative MCL cell lines Jeko-1 and Rec-1 is inhibited by greater than 80% after exposure to 20 nM bortezomib for 4 hours. This treatment decreased NF-κB activity in Jeko-1 cells, but failed to do so in Rec-1 cells when assessed by electrophoretic mobility shift assay (EMSA). Concurrently, Rec-1 cells were more resistant to the cytotoxic effects of bortezomib than Jeko-1 cells. Consistent with a proteasome inhibitor resistant pathway of activation described in mouse B-lymphoma cells (WEHI231) and a breast carcinoma cell line (MDA-MB-468), the bortezomib-resistant NF-κB activity in Rec-1 cells is inhibited by calcium chelators, calmodulin inhibitors, and perillyl alcohol, a monoterpene capable of blocking L-type calcium channels. Importantly, the combination of perillyl alcohol and bortezomib is synergistic in eliciting Rec-1 cell cytotoxicity. The relevance of these results is illuminated by the additional finding that a considerable fraction of primary MCL samples (8 out of 10) displayed bortezomib-resistant constitutive NF-κB activity.

**Conclusion:**

Our findings show that bortezomib-resistant NF-κB activity is frequently observed in MCL samples and suggest that this activity may be relevant to MCL biology as well as serve as a potential therapeutic target.

## Background

Mantle cell lymphoma (MCL) is an aggressive B-cell non-Hodgkin lymphoma genetically characterized by the t(11;14)(q13;q32) translocation with overexpression of cyclin D1. [1] It typically presents as advanced disease in men over 60 years of age and accounts for approximately 5% of all non-Hodgkin lymphoma (NHL) with an incidence of approximately 3,000 cases per year in the United States [2-5]. MCL remains a therapeutic challenge, having the worst 5-year survival of any lymphoma subtype in the NHL classification project and a median survival of only 3 or 4 years [6-15].

The need for new treatment strategies has led to the development of a novel class of pharmacologic agents, the proteasome inhibitors. After demonstrating activity in patients with refractory multiple myeloma with a manageable toxicity profile, bortezomib (Velcade; Millenium Pharmaceuticals Inc, Cambridge, MA), a reversible inhibitor of chymotryptic-like activity in the 26S proteasome, was the first agent of this class to be approved for clinical use [16-20]. When administered as a single agent in relapsed MCL, response rates of 29–48% have been demonstrated [21-24]. The efficacy of bortezomib in treating previously refractory cases of MCL is believed to involve multiple signaling pathways, and among them, the nuclear factor κB (NF-κB) pathways [25-27].

NF-κB is a family of related transcription factors including NF-κB1 (p50 and its precursor p105), NF-κB2 (p52 and its precursor p100), p65 (RelA), RelB, and cRel that are typically found as homo- or heterodimers. This family of transcription factors is unique in its capacity to coordinate transcription of a diverse array of genes, including those involved in cell proliferation and resistance to apoptosis, thereby opening avenues for malignant cell growth [28-31]. NF-κB has been implicated as an important mediator of malignant cell growth and survival in MCL. While most non-malignant cells contain inactive NF-κB complexes sequestered in their cytoplasm, constitutive activation of NF-κB has been frequently observed in both MCL cell lines and primary MCL samples and accordingly, inhibition of this constitutive activation has been shown to elicit cell cycle arrest and cell death [25,26,32,33].

There are multiple distinct signaling pathways that lead to activation of NF-κB. The best characterized mechanisms of inducible NF-κB activation are termed "canonical" and "non-canonical". The canonical pathway is initiated through receptor binding by ligands such as tumor necrosis factor alpha or lipopolysaccharide. This activates a signaling cascade involving the phosphorylation of the NF-κB inhibitor, IκBα, by the IκB kinase (IKK) complex, followed by its ubiquitination and subsequent degradation by the 26S proteasome. Degradation of IκBα allows NF-κB to translocate to the nucleus where it can regulate transcription [29,34]. Alternatively, the non-canonical pathway is activated by ligands such as B-cell activating factor family or lymphotoxin beta leading to the NF-κB inducing kinase (NIK) and IKK alpha (IKKα) facilitated, proteasome-mediated, conversion of p100 to its active form, p52 [35-37]. This ultimately results in selective activation of the p52/RelB complex and activation of its target gene. There are also other mechanisms of NF-κB activation including those that involve the calpain family of calcium-dependent proteases [38-40], hypoxia-reoxygenation-induced tyrosine phosphorylation of IκBα that leads to its dissociation from NF-κB without degradation [41], ultraviolet irradiation induced phosphorylation of IκBα via casein kinase II leading to its degradation and NF-κB activation [42], and a pathway referred to as PIR (proteasome inhibitor resistant) that involves degradation of IκBα in a manner independent of proteasomes but dependent on calcium, calmodulin, and L-type calcium channels [43-46]. Thus, depending on physiologic and pathologic settings, different NF-κB activation mechanisms could be involved, some sensitive to proteasome inhibitors while others not.

While inducible pathways of NF-κB activation are better characterized, less is known about the mechanisms of constitutive NF-κB activation. In addition to MCL, constitutive NF-κB activity has also been demonstrated in other malignancies including, adult T-cell leukemia, diffuse large B-cell lymphoma, multiple myeloma, Reed-Sternberg cells of Hodgkin lymphoma, pancreatic adenocarcinoma, prostate adenocarcinoma, squamous cell carcinoma of the head and neck, and breast carcinoma [47-53]. Recent studies examining multiple myeloma samples have demonstrated that ~20% of patient samples harbor mutations or amplifications of genes whose protein products participate in either canonical or non-canonical NF-κB signaling pathways, leading to constitutive NF-κB activation [54,55]. Among the many different NF-κB activation mechanisms, the PIR pathway has been found constitutively active in a variety of cell lines analyzed, including the WEHI231 and other murine B-cell lymphoma lines, during in vitro differentiation of a v-Abl transformed pre-B cell line, the MDA-MB 468 human breast cancer cell line, and the RPMI8226 human multiple myeloma cell line [43-46,56-59]. Importantly, the PIR pathway for constitutive NF-κB activation has been shown to be highly resistant to over 10 different types of proteasome inhibitors analyzed, including bortezomib, but selectively sensitive to inhibition by perillyl alcohol (POH), a monoterpene with anticancer activity which can, among other activities, block L-type calcium channels [45,46,58,60]. It is as yet unknown whether MCL harbors PIR constitutive NF-κB activity.

Herein, we show that Rec-1 cells have constitutive PIR NF-κB activation and they are more bortezomib-resistant relative to Jeko-1 cells which lack PIR activation. Inhibiting PIR NF-κB activity with POH is synergistic with bortezomib in inducing Rec-1 cell death. Moreover, eight of ten primary MCL samples analyzed demonstrated constitutive NF-κB activity with high resistance to bortezomib. Overall, we show that bortezomib-resistant constitutive NF-κB activity is frequently observed in primary MCL samples, suggesting that nonproteasome-dependent mechanisms, including the PIR pathway, may be relevant to MCL biology.

## Materials and methods

### Cell lines, antibodies, and chemicals

Two well-characterized Epstein-Barr virus-negative human MCL cell lines, Jeko-1 and Rec-1 [61] were cultured in RPMI 1640 (Mediatech Inc, Herndon, VA) supplemented with 10% fetal bovine serum (HyClone Laboratory, Logan, UT), 1250 U of penicillin G (Sigma-Aldrich, St. Louis, MO), 0.5 mg of streptomycin sulfate (Sigma), and 0.1% Basal Medium Eagle amino acid supplement in 37°C incubators with 5% CO_2_. p65 (C20) and RelB (C19) antibodies were purchased from Santa Cruz Biotechnology (Santa Cruz, CA), p50 (06–886) and p52/p100 (06–413) antibodies were purchased from Upstate Biotechnologies (Lake Placid, NY), and the cRel (SA-172) antibody was purchased from Biomol (Plymouth Meeting, PA). Bortezomib was commercially obtained from Millenium Pharmaceuticals, Inc. (Cambridge, MA) for experimental purposes. Propiduim iodide (PI) was purchased from Sigma-Aldrich (St. Louis, MO) and Aldrich Chemical Company (Milwaukee, WI) supplied the perillyl alcohol (POH). BAPTA/AM [bis-(*o*-aminophenoxy)ethane-*N, N, N', N'*-tetra-acetic acid tetrakis-(acetoxymethyl ester)], EGTA/AM [ethylene glycol tetra-acetic acid tetrakis-(acetoxymethyl ester)], W12 [*N*-(4-aminobutyl)-1-naphthalenesulphonamide], and W13 [N-(4-aminobutyl)-5-chloro-1-naphthalenesulphonamide] were purchased from Calbiochem (San Diego, CA).

### Electrophoretic mobility shift assay (EMSA)

For MCL cell lines, approximately 1.0 × 10^6 ^cells/mL were allowed to rest for 2–4 hours in a 5% CO_2 _incubator at 37°C. Cells were then treated with the appropriate chemicals and spun down at 13,000 × *g *in a benchtop Eppendorf centrifuge for 10 seconds. Pellets were washed once in 1× phosphate buffered saline (PBS), spun down again at 13,000 × *g *in a benchtop Eppendorf centrifuge for 10 seconds, and stored at -70°C. Cell pellets were lysed in totex buffer (20 mM HEPES [pH 7.9], 350 mM NaCl, 20% glycerol, 1% NP-40, 1 mM MgCl_2_, 0.5 mM EDTA, 0.1 mM EGTA, 0.5 mM DTT, 0.5 mM PMSF, 1 ug/mL aprotinin), spun down at 13,000 × *g *in an Eppendorf centrifuge for 10 minutes at 4°C to remove cell debris, and the supernatant subjected to the assay as described previously [62], except for the use of a mini-EMSA tailored for the limited protein extracts from the primary MCL samples. Here, 6 μL of reaction was set up and 5 μL of each reaction mix was loaded onto 10 mm × 3 mm × 1 mm wells. The Igκ-κB oligonucleotide probe was as described previously [62]. For supershift assays, the indicated antibodies were added to each reaction prior to the addition of the Igκ-κB probe. Gels were dried and exposed on Phosphor Screens (Amersham Biosciences, Piscattaway, NJ) followed by quantitation of NF-κB DNA-binding through ImageQuant analysis (GE Healthcare, Piscattaway, NJ). Fold intensity for each lane was normalized to Oct-1 values from the same sample and then to the vehicle treated control values at the indicated time point.

For primary MCL samples, the equivalent of one half of the protein from 5.0 × 10^4 ^cells from the Z138 MCL cell line was loaded in each well of the mini-EMSA. In order to correct for differences in amounts of protein between each lane in a gel, fold intensity for each lane was normalized to Oct-1 values from the same sample and then to the vehicle treated control value. Additionally, one half of total protein from 5.0 × 10^4 ^Z138 cells was run in parallel with every patient sample to serve as an internal standard in order to facilitate comparison of relative constitutive NF-κB activities between each of the MCL patient samples. To this end, densitometry values of primary MCL samples were additionally normalized to the internal standard Z138 value.

### Apoptosis assays

PI staining was used to examine cell death in non-fixed cells by flow cytometric analysis. 30 ug/mL of PI was added to the cells 5 minutes prior to data collection on a FACSCalibur flow cytometer (Becton Dickinson Bioscience, San Jose, CA) followed by analysis on Cell Quest software (Becton Dickinson Bioscience) [63].

### 26S proteasome activity assay

A live-cell luminogenic substrate based assay of the chymotrypsin-like activity of the 26S proteasome was used according to the manufacturer's instructions (Proteasome-Glo Assay with Suc-LLVY-Glo substrate, Promega Corporation, Madson, WI). Luminescence was recorded on a plate-reading luminometer (PerkinElmer, Waltham, MA).

### Drug combination evaluation

The cytotoxic effect of combined bortezomib and POH on MCL cell lines was determined by the combination index (CI) method, based on the median-effect principle derived by Chou and Talalay [64,65], and analyzed by the Calcusyn computer program (Biosoft, Cambridge, UK). Briefly, median-effect analysis is a measure of synergism or antagonism where the median-effect equation correlates the drug dose with cytotoxic effect in the equation: *f*_*a*_/*f*_*u *_= (*D/D*_*m*_)^*m *^in which *D *is the dose of the drug; *D*_*m *_is the median-effect dose signifying the potency, determined from the x-intercept of the median-effect plot; *f*_*a *_is the fraction affected by the dose; *f*_*u *_is the fraction unaffected; and *m *is an exponent that signifies the sigmoidocity of the dose-effect curve. CI is then calculated: CI = (*D*)_1_/(*D*_*x*_)_1 _+ (*D*)_2_/(*D*_*x*_)_2_, where (*D*)_1 _and (*D*)_2 _are the doses of drug 1 and drug 2 that have *x *effect when used in combination and (*D*_*x*_)_1 _and (*D*_*x*_)_2 _are the doses of drug 1 and drug 2 that have the same *x *effect when used alone. Synergy is present when the CI is less than 1.0. The combination is additive when CI equals 1.0, and is antagonistic when greater than 1.0.

### Primary MCL samples

Twenty samples of fresh bone marrow, peripheral blood, or lymph node (Table 1) were obtained from patients diagnosed with MCL according to World Health Organization criteria [1] either at the time of initial diagnosis or disease recurrence. The specimens were cryopreserved in a solution containing 20% DMSO in RPMI media with 10% fetal calf serum after enrichment of mononuclear cells by density gradient separation and stored at -140°C. At the time of analysis, specimens were thawed and anti-CD19 magnetic microbeads were used to positively select cells through the MACS cell sorting system following the manufacturer's protocol (Miltenyi Biotec, Auburn, CA). From the 20 cases, 10 that had greater than 60% lymphocytes that co-expressed CD5 with CD19 and had greater than 5.0 × 10^5 ^cells for EMSA analysis were included. In these 10 cases, the mean percentage of lymphocytes demonstrating the MCL phenotype with combined expression of CD5 and CD19 was 78% (range 62% to 96%) as analyzed on a FACSCalibur flow cytometer (Becton Dickinson, Franklin Lakes, NJ) with anti-CD5-PE and anti-CD19-PECy5 antibodies (BD Biosciences, San Jose, CA) (Table 1). The primary cells were cultured in media identical to that used for the MCL cell lines.

**Table 1:** Characteristics of MCL patient samples.

**Patient no.**	**Age (years)**	**Gender**	**Source**	**Disease status**	**Morphologic variant**	**Tumor cells (%)**^**1**^
1	81	F	Bone marrow	Primary	Blastic	61.6
2	58	F	Bone marrow	Recurrence	Classic	95.6
3	77	F	Peripheral blood	Primary	Blastic	74.8
4	75	F	Bone marrow	Primary	Blastic	71.1
5	56	F	Lymph node	Primary	Classic	83.0
6	53	F	Lymph node	Recurrence	Classic	88.6
7	49	M	Peripheral blood	Primary	Classic	96.1
8	57	F	Bone marrow	Primary	Classic	83.6
9	62	M	Bone marrow	Recurrence	Classic	76.8
10	67	M	Bone marrow	Primary	Classic	69.7

^1 ^Percentage of cells co-expressing CD5 and CD19 after CD19 positive cell selection.

## Results

### Constitutive NF-κB activation in two MCL cell lines is associated with distinct NF-κB heterodimers and mechanisms

**Figure 1:**
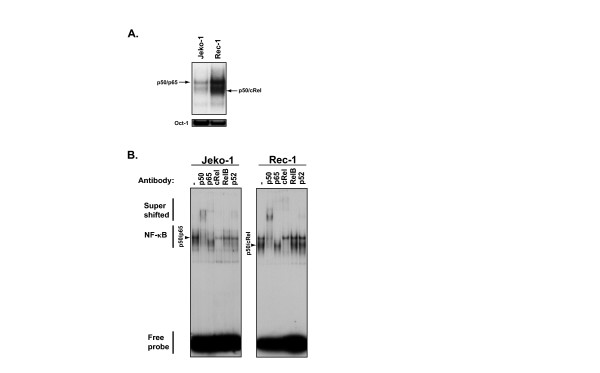
**Constitutive NF-κB activation in MCL cell lines**. (A) Electrophoretic mobility shift assay (EMSA) of whole cell extracts from 1.0 × 10^6 ^cells of EBV-negative MCL cell lines probed with ^32^P-radiolabeled oligonucleotide containing the consensus NF-κB binding sequence. Oct-1 binding is shown as a loading control. Assignment of the NF-κB complexes are based on supershift analysis shown in (B). (B) Supershift EMSA of whole cell extracts from MCL cell lines.

Constitutive activation of NF-κB has been implicated as a critical modulator of malignant cell growth and survival in a number of human malignancies including MCL [25,47-53]. To avoid complications arising from the latent membrane protein 1 of EBV, which has been shown to activate NF-κB [66], we first investigated constitutive NF-κB complexes in the EBV-negative MCL cell lines, Jeko-1 and Rec-1, using EMSA. Both cell lines demonstrated constitutive NF-κB activation with Rec-1 cells having relatively more activity than Jeko-1 cells (Figure 1A). Supershift analysis showed that Jeko-1 and Rec-1 cells contain both p50/p65 and p50/cRel heterodimers, with Rec-1 having relatively more p50/cRel (Figure 1B). NF-κB p50/p65 and p50/cRel heterodimers can be activated via both canonical, PIR, and other pathways [34,44,45], suggesting that distinct mechanisms mediating constitutive NF-κB activation may exist in these MCL cell lines.

**Figure 2:**
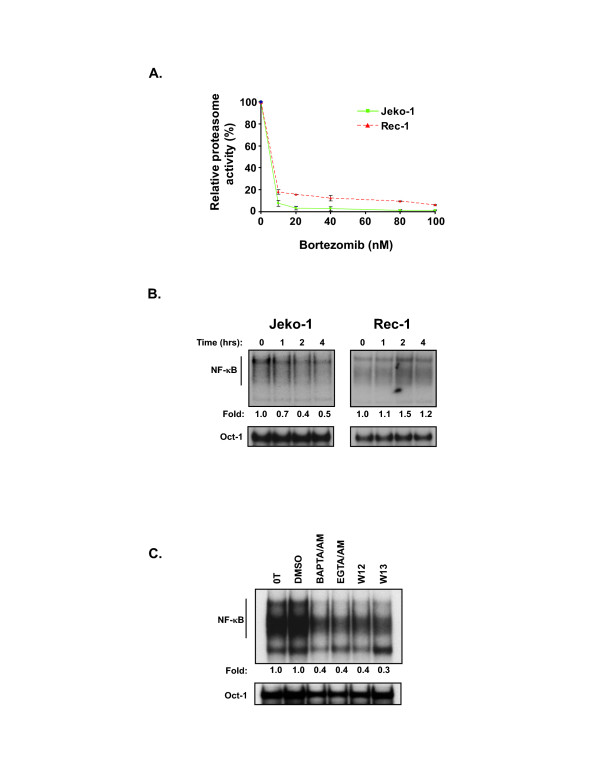
**Bortezomib-resistant constitutive NF-κB activity in Rec-1 cells**. (A) Chymotrypsin-like activity of the 26S proteasome in cell lines after exposure to increasing doses of bortezomib for 4 hours was assessed by luminescence generated by substrate cleavage (Proteasome-Glo Assay, Promega Corporation, Madison, WI). Results (mean ± 1SD from triplicate wells) are shown as a percent of luminescence relative to vehicle treated controls. (B) EMSAs of MCL cell lines treated with 20 nM bortezomib for 1 to 4 hours probed with ^32^P-radiolabeled oligonucleotide containing either the consensus NF-κB or Oct-1 binding sequence. Fold intensity was calculated by ImageQuant analysis of Phosphor Screens normalized to Oct-1 values from the same sample and then to the vehicle treated control values at the indicated time point. Gels shown are representative of one of three independent experiments. (C) EMSA of Rec-1 cells at time zero (0T) and treated with 0.1% DMSO (DMSO) for 3 hours, the calcium chelators BAPTA/AM (60 μM) and EGTA/AM (60 μM) for 3 hours, or the calmodulin inhibitors W12 (40 μM) and W13 (10 μM) for 3 hours. Fold activity refers to NF-κB binding normalized to Oct-1 binding in each condition. Gel shown is representative of 3 independent experiments.

We next investigated whether a proteasome inhibitor resistant activation pathway was involved in maintaining constitutive NF-κB activity in Jeko-1 and Rec-1 cells. To evaluate the efficacy of inhibition of the chymotryptic-like proteasomal activity in these cells by bortezomib, a live-cell luminogenic substrate based assay was used (see Materials and Methods). Both cell lines showed marked inhibition of proteasomal activity following treatment with bortezomib. Exposure of these cells to 20 nM of bortezomib for four hours decreased proteasome activity to less than 20% of vehicle treated controls (Figure 2A).

**Figure 3:**
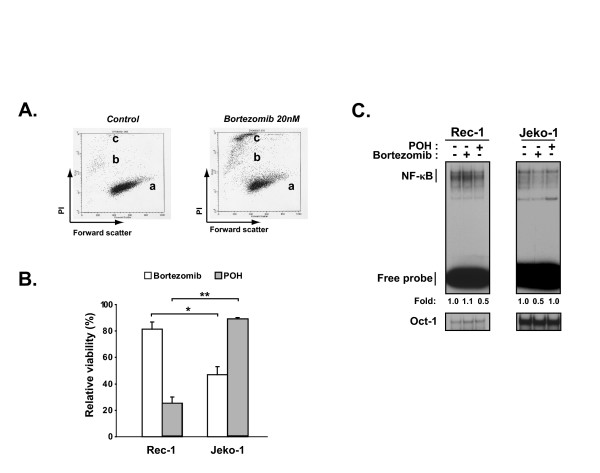
**Cytotoxic effect of bortezomib and POH on MCL cell lines**. (A) Assessment of viability by staining non-fixed Rec-1 cells with PI (30 μg/mL) followed by flow cytometric analysis showing populations of (a) live cells, (b) necrotic cells, and (c) apoptotic cells. (B) Viability of Rec-1 and Jeko-1 cells treated with either 20 nM bortezomib or 0.3 mM POH for 24 hours relative to controls exposed only to vehicle shown as mean ± 1SD from triplicate wells. The unpaired *t*-test between viability of Jeko-1 and Rec-1 cells treated with bortezomib * *p *= 0.002 or POH **p = 0.0001 were both statistically significant (*p *< 0.01). (C) EMSAs of whole cell extracts from 1.0 × 10^6 ^cells treated with 20 nM bortezomib or their respective median-effect dose of POH for 4 hours probed with ^32^P-radiolabeled oligonucleotide containing either the consensus NF-κB or Oct-1 binding sequence. Fold activity refers to NF-κB binding normalized to Oct-1 binding in each condition.

Next, we evaluated the effect of proteasome inhibition on constitutive NF-κB activity. NF-κB activity was decreased in Jeko-1 cells but not in Rec-1 cells after 4 hour treatment with either 20 nM bortezomib (Figure 2B) or 100 nM of bortezomib (data not shown). The bortezomib-resistance of constitutive p50/p65 and p50/c-Rel activity in Rec-1 cells could be due to the involvement of the PIR pathway. To determine if this is the case, we treated Rec-1 cells with agents known to block the PIR pathway, including calcium chelators (BAPTA/AM and EGTA/AM), calmodulin inhibitors (W12 and W13) and the anticancer agent, POH [43-46]. As shown in Figure 2C and 3C, these agents effectively blocked constitutive NF-κB activity in this cell line, thereby suggesting the involvement of the PIR pathway.

### Synergism of POH with bortezomib in a MCL cell line harboring bortezomib-resistant constitutive NF-κB activity

Because NF-κB has been shown to mediate cellular growth and survival in MCL [25], we evaluated whether cells possessing bortezomib-resistant constitutive NF-κB activity would in fact demonstrate a survival advantage in the face of bortezomib treatment. PI staining was used to assess the cytotoxic effect of 24 hour treatment with 20 nM bortezomib on the MCL cell lines. This method enables enumeration of both apoptotic and necrotic non-fixed cells by flow cytometric analysis (Figure 3A) [46,63]. Jeko-1 cells were sensitive to bortezomib induced cytotoxicity and Rec-1 cells were significantly less so with 47 ± 6.2% and 82 ± 5.1% viability respectively, relative to vehicle treated controls (Figure 3B).

**Figure 4:**
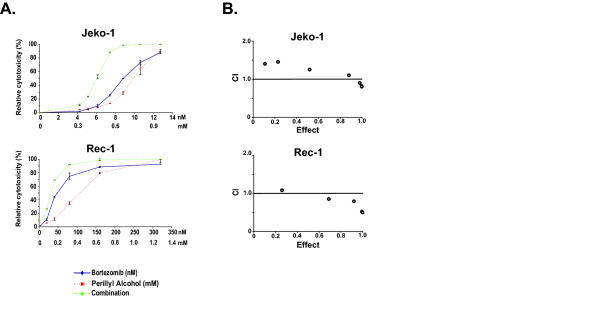
**POH synergizes with bortezomib in inducing cytotoxicity in Rec-1, but not Jeko-1 cells**. (A) Viability assessed by PI staining with flow cytometric quantitation relative to controls exposed only to vehicle at various doses of bortezomib, POH, or combination of both for 24 hours. Results expressed as mean ± 1SD of triplicate wells. (B) Combination index (CI) plots generated by the computer software CalcuSyn (Biosoft, Cambridge, UK) according to the Chou-Talalay equation ("Materials and Methods") from cytotoxicity data shown in (A). Effect is equivalent to cytotoxicity where effect of 1.0 = 100% relative cytotoxicity. Synergy is present when CI < 1.0. The combination is additive when CI = 1.0, and antagonistic when CI > 1.0.

To determine if inhibition of the PIR NF-κB activity with POH would induce bortezomib sensitization, we next evaluated synergy between bortezomib and POH in the Rec-1 cells. We also treated Jeko-1 cells as a negative control since constitutive NF-κB activity in this cell line demonstrated high sensitivity to bortezomib but not to POH (Figure 3C). To accurately compare the effect of POH on NF-κB activity in Jeko-1 and Rec-1 cells, we first determined the potency of POH in each cell line by assessing cytotoxicity through PI staining followed by flow cytometric quantification and calculation of the median-effect dose (D_*m*_) for each cell line (see methods). We found that POH was more potent in Rec-1 (D_*m *_= 0.32 mM) than Jeko-1 (D_*m *_= 0.67 mM) cells (Figure 4A). These anti-MCL doses are similar in range to those seen against other cancer types [67-70]. When treated with their respective D_*m *_of POH for 4 hours, NF-κB activity was reduced in Rec-1 but not Jeko-1 cells (Figure 3C). When Rec-1 cells were treated with a fixed ratio of bortezomib and POH, analysis for synergy by the method of Chou-Talalay showed combination indices (CI) of 0.83 and 0.74 at ED75 and ED90, respectively (Figure 4B). These CI values are <1.0, indicating synergistic toxicity with POH and bortezomib. In contrast, parallel synergy experiments in Jeko-1 cells demonstrated CIs of 1.13 and 1.03 at ED75 and ED90, respectively (Figure 4B), suggesting not only a lack of synergy, but even a modest antagonism. These findings show that inhibition of constitutive PIR NF-κB activity in Rec-1 cells by POH induces synergistic toxicity with bortezomib. This synergy is not seen in Jeko-1 cells that harbor constitutive NF-κB activity utilizing the proteasome-dependent canonical pathway.

### Bortezomib-resistant constitutive NF-κB activity is frequently observed in primary MCL samples

**Figure 5:**
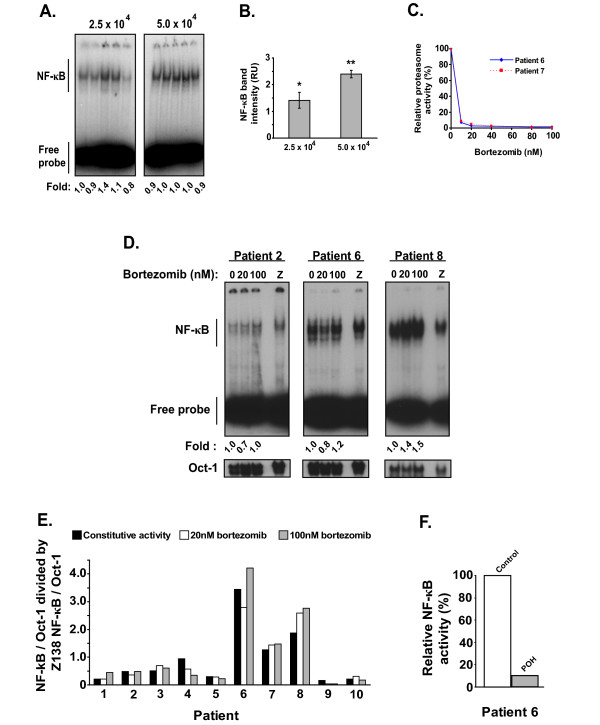
**Bortezomib-resistant constitutive NF-κB activity in MCL patient samples**. (A) Mini-EMSA performed on one half of the protein extracts from 2.5 × 10^4 ^and 5.0 × 10^4 ^Z138 cells probed with ^32^P-radiolabeled oligonucleotide containing the consensus NF-κB sequence. (B) Mean ± 1SD of densitometric values in relative units (RU) from ImageQuant analysis of Phosphor Screens exposed to mini-EMSA from (A). *Coefficient of variation (CV) = 20.9% **CV = 5.9% (C) Chymotrypsin-like activity of the 26S proteasome in 2 patient samples after exposure to increasing doses of bortezomib for 4 hours as assessed by luminescence generated by substrate cleavage (Proteasome-Glo Assay, Promega Corporation, Madison, WI). Results are shown as percent of luminescence relative to a vehicle treated control in a single experiment. (D) Mini-EMSA of anti-CD19 magnetic microbead selected cells from MCL patient samples treated with 20 nM and 100 nM of bortezomib for 4 hours. Z represents a standard composed of 5.0 × 10^4 ^Z138 cells run with each patient sample as an internal control. The protein extract from this standard was halved, with each half run alongside corresponding halved patient samples, where one half was probed with oligonucleotide containing the NF-κB consensus binding sequence and the other half probed with oligonucleotide containing the Oct-1 consensus binding sequence. (E) NF-κB DNA-binding of MCL patient samples corrected for Oct-1 intensity then normalized to their respective vehicle treated control and finally normalized to Z138 internal control for MCL patient samples processed and analyzed as described in (D). (F) NF-κB DNA-binding of MCL patient 6 after treatment with 1.0 mM POH for 4 hours relative to vehicle treated control, corrected for Oct-1 intensity.

Since the number of primary MCL cells in patient-derived samples is very limited in general, we next developed a scaled down version of the traditional EMSA assay ("mini-EMSA") (see methods). In order to validate the precision of this assay, assessment of NF-κB band intensities of 5 replicate samples calculated by ImageQuant analysis of Phosphor Screens exposed to the dried mini-EMSAs loaded with half the protein extracts from 2.5 and 5.0 × 10^4 ^cells from the Z138 MCL cell line was performed. The other half samples were used for the loading control Oct-1 binding. The coefficient of variation of this assay for 5.0 × 10^4 ^Z138 cells was of 5.9% (Figure 5A and 5B). While cell numbers greater than 5.0 × 10^4 ^produced reproducible results, when cell numbers were reduced below this number, the reproducibility suffered greatly. Thus, we used 5.0 × 10^4 ^Z138 cell equivalents as the target amount of protein from patient samples to load in each gel. We also ran Z138 cell samples as a standard in each EMSA so that we could compare results between primary samples run on different days in spite of day to day variances of the specific activity of the Igκ-κB probe (Figure 5D). Constitutive NF-κB activity was present in all primary samples and ranged in intensity from 0.16 to 3.45 fold of the Z138 standard cells (Figure 5E).

Next, we investigated the sensitivity of constitutive NF-κB activity in primary MCL samples to treatment with 20 nM or 100 nM of bortezomib for four hours. The degree of proteasome inhibition achieved at these dosages in two representative patients (patient 6 and patient 7) was comparable to that seen in the MCL cell lines tested by the same assay (Figure 5C). Relative to vehicle treated controls, only two patients (patient 4 and patient 9) demonstrated a 50% or greater decrease of NF-κB DNA-binding after treatment by either dose of bortezomib (Figure 5E). Constitutive NF-κB activity in patients 2 and 6 showed a very modest inhibition with 20 nM bortezomib, but interestingly not at the 100 nM dose. The others did not show any overt inhibition of NF-κB activity. Some patient samples (3, 7 and 8) showed modest increases in constitutive NF-κB activity after treatment of cells with bortezomib. Significantly, POH treatment of cells from patient 6, which had the most robust degree of constitutive activity, resulted in marked inhibition of NF-κB activity (Figure 5F). These results reveal that constitutive NF-κB activity present in a considerable fraction of primary MCL cases is refractory to inhibition by high dose bortezomib treatment. Moreover, this resistance is at least in part due to the involvement of the PIR pathway in MCL samples.

## Discussion

Under normal circumstances, the NF-κB signaling pathway is tightly controlled and plays important roles in lymphocyte development and the immune response; however, dysregulation of this pathway can lead to its constitutive activation in cancer cells. Although the proteasome inhibitor bortezomib can target the constitutive NF-κB activity maintained by both canonical and non-canonical pathways, proteasome inhibitor resistant mechanisms of NF-κB activation have been identified and one of them in particular, the PIR pathway, is constitutively activated in several cancer cell lines [43-46,56-58]. The PIR pathway may provide cancer cells a mechanism of maintaining constitutive NF-κB activation in the face of proteasome inhibition and confer a degree of relative resistance to proteasome inhibitor induced cytotoxicity. In turn, assessing the prevalence of bortezomib-resistant constitutive NF-κB activity in MCL patient samples illuminates whether proteasome inhibition can efficiently abrogate constitutive NF-κB activity MCL patients. Here, we found that bortezomib failed to decrease NF-κB activity by more that 50% in eight of ten primary patient samples, despite treatment with doses that elicited greater than 80% proteasome inhibition, which is the clinical target with this drug. These results demonstrate a surprising finding that constitutive NF-κB activity in primary MCL cells is often resistant to bortezomib exposure, at least *in vitro*.

We evaluated the EBV-negative Jeko-1 and Rec-1 cell lines to eliminate the effect of NF-κB activation by latent membrane protein 1, which would work via the canonical pathway [66]. Dosages of bortezomib chosen were reflective of pharmacokinetic studies where patients on standard bortezomib dosing regimens of 1.0 mg/m^2 ^had estimated peak plasma concentrations of approximately 200 nM that rapidly decreased and plateaued at approximately 5 nM in less than 5 hours, and concordantly, peak inhibition of proteasome activity in whole blood lysates reached approximately 75%, falling off to approximately 35% after 24 hours [71-73]. As such, in the MCL cell lines, we used a 20 nM dose of bortezomib for 4 hours that produced at least 80% proteasome inhibition to reflect maximal proteasome inhibition achievable with a reasonably attainable plasma bortezomib concentration. In primary MCL samples, because we could not assess the effect of bortezomib on proteasome activity in each case due to limitation of MCL cell number, we treated samples with both 20 nM and 100 nM bortezomib when determining its effect on NF-κB activation to ensure that sufficient proteasome inhibition was attained.

Constitutive NF-κB activity was present in both Jeko-1 and Rec-1 cells. Jeko-1 cells appeared to constitutively activate NF-κB through the canonical pathway as evidenced by the presence of largely p50/p65 heterodimers and inhibition of constitutive NF-κB activity by bortezomib. In contrast, Rec-1 cells appeared to activate NF-κB through the PIR pathway, where despite proteasome inhibition by bortezomib, DNA-binding by largely p50/cRel heterodimers was not inhibited; similar to our observations in the WEHI231 murine B-cell lymphoma cell line which displays a high-level of proteasome inhibitor resistant constitutive NF-κB activity [43-46,56-58]. This activity was nevertheless sensitive to inhibition by calcium chelators, calmodulin inhibitors, and POH, agents that block the PIR pathway but do not generally block canonical or non-canonical pathways.

POH is a monoterpene derived from plant essential oils that has been shown to have significant antitumor activity against multiple cancer models. [60,67-70] Its mechanism of action includes inhibition of G-protein prenylation [74,75] and alterations in cell cycle genes with decreased cyclin D1 [76,77]. Of interest, POH has also been shown to inhibit the L-type calcium channels important for the maintenance of PIR constitutive NF-κB activity in both WEHI231 lymphoma cells and MDA-MB-468 breast cancer cells [46]. Indeed, in Rec-1 cells, POH treatment conferred a marked decrease in constitutive NF-κB activity and correspondingly induced cytotoxicity. In contrast, in Jeko-1 cells that harbor constitutive NF-κB activation maintained by the canonical pathway, POH did not decreased NF-κB activity and were more resistant to POH relative to Rec-1 cells. Moreover, POH synergistically caused toxicity in Rec-1 cells, but not in Jeko-1 cells. These results demonstrated a parallelism between bortezomib versus POH sensitivity to canonical versus PIR constitutive NF-κB activation. In MCL cells that harbor constitutive PIR NF-κB activity, POH can synergize with bortezomib to cause cell death.

It is clearly simplistic to view NF-κB as the sole independent modulator of MCL or other cancer cell death phenotypes. Numerous NF-κB-independent mechanisms of bortezomib-induced apoptosis have been demonstrated in multiple myeloma, lung cancer cells, and squamous cell carcinomas. These include the induction of p53, mouse double minute 2, p21, and p27; phosphorylation of c-Jun NH(2)-terminal kinase and c-Jun; enhanced AP-1 DNA-binding; and induction of the unfolded protein response via accumulation of unfolded or misfolded proteins in the endoplasmic reticulum [78-82]. Furthermore, it has been shown in MCL that a major mode of cell death induction by bortezomib is through generation of ROS and Noxa induction that results in activation of the mitochondrial apoptotic pathway [27]. It is nevertheless also well established that NF-κB can confer additional resistance to MCL cells, although it may not be the major decisive factor in controlling the overall sensitivity to bortezomib. Since the patient samples analyzed herein did not come from those who have been treated with bortezomib and the sample size was relatively small, the correlation between the presence of proteasome inhibitor resistant constitutive NF-κB activity in MCL samples and bortezomib response in patients could not be assessed. Nevertheless, our data demonstrating synergy between bortezomib and POH (Figure 4A and 4B) suggest the possibility that targeting the PIR pathway of constitutive NF-κB activity with POH (or other anti-PIR agents) could increase the sensitivity of MCL cells *in vivo*. This is particularly relevant, since of the 10 evaluable MCL patient cases, all demonstrated constitutive NF-κB activation and only 2 cases showed greater than a 50% inhibition of NF-κB activity after exposure to bortezomib.

## Conclusion

This study demonstrates that constitutive NF-κB activity is not only present in MCL patient samples, but this activity is largely resistant to proteasome inhibition by bortezomib *in vitro*. Additionally, by using Rec-1 cells as a model of bortezomib-resistant constitutive NF-κB activity, we showed that blocking this activity with POH was synergistic with bortezomib in inducing cytotoxicity. Thus, our data suggests that the full therapeutic potential of blocking constitutive NF-κB activity as a treatment strategy for MCL has yet to be fully realized and supports efforts invested towards the discovery and development of compounds capable of inhibiting bortezomib-resistant constitutive NF-κB activity with potency and specificity superior to POH.

## Declaration of competing interests

The authors declare that they have no competing interests.

## Authors' contributions

DTY contributed to the design of the study, development of the assay system for patient samples, performance of the research, analysis and interpretation of the data, and the writing of the paper. KHY. and BSK contributed to the design of the study, acquisition of patient samples, and critical review of the manuscript. SMarkovina contributed to the initial acquisition of patient samples, development of the assay system for patient samples, and critical review of the manuscript. SMiyamoto contributed to the conceptualization and design of the study, analysis and interpretation of the data, and the writing of the paper. All authors approved the final version of the manuscript.
